# Improving the performance of the PLB index for ligand-binding site prediction using dihedral angles and the solvent-accessible surface area

**DOI:** 10.1038/srep33232

**Published:** 2016-09-13

**Authors:** Chen Cao, Shutan Xu

**Affiliations:** 1 College of Computer Science and Technology, Jilin University, Changchun, Jilin, China; 2 Key Laboratory of Symbol Computation and Knowledge Engineering of the Ministry of Education, Jilin University, Changchun, Jilin, China; 3 Department of Biochemistry and Molecular Biology, Institute of Bioinformatics, University of Georgia, Athens, GA, USA

## Abstract

Protein ligand-binding site prediction is highly important for protein function determination and structure-based drug design. Over the past twenty years, dozens of computational methods have been developed to address this problem. Soga *et al*. identified ligand cavities based on the preferences of amino acids for the ligand-binding site (RA) and proposed the propensity for ligand binding (PLB) index to rank the cavities on the protein surface. However, we found that residues exhibit different RAs in response to changes in solvent exposure. Furthermore, previous studies have suggested that some dihedral angles of amino acids in specific regions of the Ramachandran plot are preferred at the functional sites of proteins. Based on these discoveries, the amino acid solvent-accessible surface area and dihedral angles were combined with the RA and PLB to obtain two new indexes, multi-factor RA (MF-RA) and multi-factor PLB (MF-PLB). MF-PLB, PLB and other methods were tested using two benchmark databases and two particular ligand-binding sites. The results show that MF-PLB can improve the success rate of PLB for both ligand-bound and ligand-unbound structures, particularly for top choice prediction.

Proteins perform their biological functions by interacting with other molecules (ligands), such as DNA, antigens, drugs or even other proteins. Identifying the residues participating in the interaction site and characterizing the geometric features of the site are important steps for identifying these regions and understanding protein functions. Protein-DNA binding sites show the most obvious amino acid preferences because positively charged residues, such as arginine and lysine, likely face the negatively charged phosphate backbone of DNA^1^.

In this manuscript, we focus on the detection of small molecule ligand-binding sites because it is a prerequisite for protein-ligand docking and the first step of structure-based drug discovery^2^,^3^. Many protein ligand-binding site prediction methods have been developed over the past 20 years. These methods can be generally categorized into four classes: geometry-based methods, energy-based methods, sequence-based methods, and combined methods.

Geometry-based methods apply a grid or sphere to detect all cavities on the target protein surface (e.g., Ligsite, Surfnet, PASS, PocketFinder and CAST)^4^,^5^,^6^,^7^,^8^, and most of these methods assume that the ligand-binding site coincides with the largest cavity. Energy-based methods search for energetically favourably pockets for ligand binding by calculating the interaction energy between the chemical probe and protein atoms (e.g., SITEHOUND, Q-SiteFinder and FTMap)^9^,^10^,^11^; in these methods, the probe can be a methyl group or atoms, such as carbon and phosphorus. Sequence-based methods employ information from sequence conservation or homologous structures (e.g., TargetS, LigandRF and Multi-RELIEF)^12^,^13^,^14^, and homologous-based methods have been found to the most accurate by far but require structure data for close ligand-binding homologs. Most combined methods predict the ligand-binding site through two steps: First, all potential ligand-binding sites are detected by geometry-based methods, and then, the predicted ligand-binding site is determined according to the conservation of the residues in the cavity (e.g., Ligsite-csc, Surfnet-ConSurf, GalaxySite, ConCavity, and LISE)^15^,^16^,^17^,^18^,^19^. Meta-Pocket 1.0 and 2.0 (MPK1 and MPK2) combine the results of many methods to improve the prediction result^20^,^21^.

To identify a potential ligand-binding site, Soga *et al*. developed an index known as the propensity for ligand binding (PLB), which is calculated by simply summing up the RA (preference factor for an amino acid) of all residues involved in the site^22^. The PLB index has also proven to be useful for identifying druggable protein cavities^23^. The RA is derived from a database of high-quality protein-ligand complex structures and has a constant value for each type of amino acid; thus, only 20 RA values are provided^22^. However, we have found that amino acids exhibit different propensities under different solvent exposure conditions. In addition, our previous study revealed that the dihedral angles of the residues in specific regions of the Ramachandran plot reveal preferences for ligand-binding sites^24^.

In this study, a method that considers the amino acid solvent-accessible surface area (SASA) and dihedral angles based on the RA and multi-factor RA (MF-RA) was developed. In addition, similar to the PLB, the proposed MF-PLB sums up all of the amino acid MF-RAs at the ligand site. Two frequently used test datasets were applied to compare the prediction abilities of the MF-PLB, PLB, and several popular ligand-binding site prediction methods. Because large ligand-binding sites can be easily identified, we constructed two new databases to evaluate the performances of different methods for the identification of small-volume ligand-binding sites and protein-protein interface ligand-binding sites.

## Methods

### Calculating hydrogen bonds and van der Waals (*vdW*) contacts

The hydrogen bonds between a ligand and residues in a protein were calculated using the HBPLUS programme with default values^25^. Several geometrical criteria of specific atoms, including hydrogen bond donors (D) and acceptors (A), were applied for the identification of hydrogen bonds using HBPLUS. The detection of *vdW* contacts between a ligand and protein residues is simple: For a non-hydrogen ligand atom A and a non-hydrogen residue atom B, a *vdW* contact exists between the two atoms if the distance between A and B satisfies *dist*(AB)-0.5 Å < *vdW radius*(A) + *vdW radius*(B).

### SASA calculation

We applied the NACCESS programme to calculate the SASA and relative SASA for all residues^26^. NACCESS scans a probe over the *vdW* surface of the protein and calculates the SASA for each residue according to the β-centre trace of the probe. The default probe radius (1.4 Å) and behaviours (water molecules, hydrogen atoms, and HET groups are excluded from the protein structure) are used. The relative SASA describes the relative accessibility of residue X and was calculated by expressing the actual SASA of X as a percentage of the residue in an extended tripeptide: ALA-X-ALA.

### Databases

Set **N**: We constructed a non-redundant database, set **N**, consisting of 6,635 ligand-bound structures obtained from the Binding MOAD released in 2014 27 to derive the MF-RA. Binding MOAD groups structures at the 90% sequence identity level, and all of the structures in Binding MOAD having more than 70% sequence identity with any protein in set **T** or set **S** were excluded from set **N**.

Set **T** and set **S**: Set **T** and set **S** are two benchmark protein structure databases that were used to evaluate the performances of the different ligand-binding site prediction methods. The two test databases have been widely used in previous studies of ligand-binding site prediction methods^15^,^19^,^20^,^28^. Set **T** consists of 210 ligand-bound protein structures, whereas set **S** includes 96 structures that can be grouped into two classes, specifically 48 ligand-unbound protein structures and their corresponding ligand-bound forms. To obtain the actual ligand-binding sites of the 48 ligand-unbound structures, the ligand-bound structures were aligned with their ligand-unbound forms using the PyMOL align function, and the ligands’ coordinates and connectivity information were obtained from the ligand-bound structures^29^.

Set **L**: The average molecular weight of the ligands in set **S** is as high as 269 dalton, whereas that of some ligands, such as “NAD” and “HEM”, exceeds 500 dalton. Additionally, large ligand-binding sites can be easily identified by geometry-based methods or even by eye based on the three-dimensional (3D) protein structures. Set **L** was constructed to evaluate the performances of different methods for small-volume ligand-binding site prediction and consists of 169 ligand-bound structure chains downloaded from the Protein Data Bank (PDB)^30^. In the PBD, each structure chain has only one ligand, and the molecular weight of the ligand should be less than 150 dalton. Inorganic molecules and metals in protein structures are ignored, the ligand should not be completely exposed to the solvent, and the ligand-binding site is formed by the only chain in the structure. In set **L**, no two structures have more than 70% sequence identity. Detailed information for set **L** is provided in Table S6.

Set **P**: Set **P** was derived from a database of dimeric protein complexes that consists of 1,611 structures obtained in previous studies^31^,^32^. No two chains from different protein complexes share a sequence identity of 35% or higher. In addition, all ligands in proteins should be located at protein-protein interfaces: the distance between a ligand and both protein chains, defined as the shortest distance between any ligand atom and any residue atom that belongs to the protein chains, is less than 5 Å. After refinement, set **P** includes 149 protein structures and was constructed to evaluate the accuracy of the prediction of ligand-binding sites on protein-protein interaction region. Detailed information for set **P** is provided in Table S7.

### Ligsite-csc, Surfnet, ConCavity, MPK2, Q-SiteFinder and LISE prediction results

To compare the MF-PLB with other prediction methods, we downloaded the prediction results obtained with Ligsite-csc from http://projects.biotec.tu-dresden.de/pocket/15, those obtained using Surfnet, ConCavity, MPK2, and Q-SiteFinder from http://projects.biotec.tu-dresden.de/metapocket/20, and those obtained using SiteHound from http://scbx.mssm.edu/sitehound/ (using methyl carbon as the probe)^9^. We used three Python scripts, which are shown in Tables S1, S2, and S3, to upload the pdb files, set the parameters from the three above-mentioned web servers and download the prediction results automatically. The LISE Perl script was obtained from http://lise.ibms.sinica.edu.tw^19^, and PSI-BLAST was used to compute the PSSM files^33^. The newest non-redundant protein sequence databases (04/23/2016) required by PSI-BLAST were downloaded directly from ftp://ftp.ncbi.nlm.nih.gov/blast/db/ 34. The files of pocket grids calculated by Ligsite-cs were also downloaded from http://projects.biotec.tu-dresden.de/metapocket^15^.

**Figure 1:**
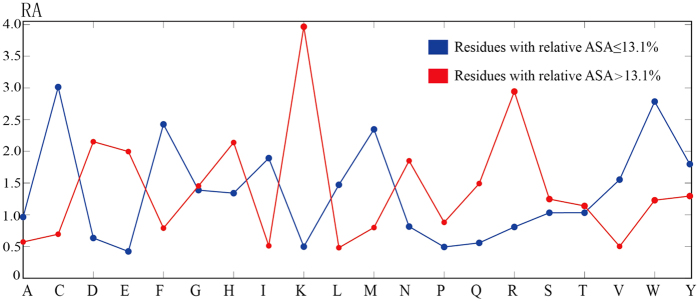
MF-RA for 20 amino acids.

The residues show different preferences for ligand-binding sites, as determined by dividing the residues into two groups, namely high-accessibility residues (relative SASA 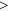
13.1%) and low-accessibility residues (1% 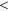 relative SASA 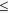 13.1%; please note that 13.1% is the median of the relative SASA values of all ligand-binding site residues in set **N**). As shown in Fig. 1, with the exception of Gly, Ser and Thr, the amino acids have significantly different RA values (preference factor, defined by Soga *et al*.). For instance, Cys has a constant RA of 1.65 according to Soga *et al*.^22^, whereas the RA values for high-accessibility and low-accessibility Cys residues are 3.01 and 0.69, respectively. As a result, dividing amino acids into two groups according to their solvent exposure can better reflect their ligand-binding site preferences.

The amino acid SASA and the dihedral angles of the amino acid backbone influence the ligand-binding site preferences. Thus, these two factors were considered, and the MF-PLB was defined as


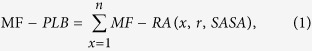


For each cavity, the MF-PLB can be obtained by simply summing the MF-RA(*x, r*, *SASA*) of all of the residues involved in the cavity. Here, *x* is the amino acid type, *r* is the Ramachandran plot region where the dihedral angle of the residue’ backbone lies (the classification of the dihedral angles is shown in Table S4), and SASA indicates the accessibility of the residue, i.e., high or low. 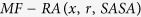 was calculated by taking the ratio of the frequency of the amino acid *x* in the protein ligand-binding site to its frequency on the protein surface. The detailed formula for the calculation of MF-RA(*x, r*, *SASA*) is shown below:


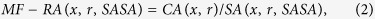


In this equation, 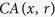 is the frequency of amino acid *x* observed at the ligand-binding site and is calculated as


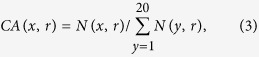


where *N*(*x, r*) and *N*(*y, r*) denote the numbers of amino acids *x* and *y* detected in the ligand-binding site, respectively; additionally, the dihedral angles of both amino acids are in the same Ramachandran region *r*. Similarly,


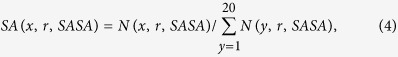


where *N*(*x, r*, *SASA*) and *N*(*y, r*, *SASA*) are the number of amino acids *x* and *y*, respectively, and the dihedral angles of both amino acids *x* and *y* should be located in the same region *r* and have the same accessibility state. In conclusion, CA is the rate of occurrence of amino acid *x* in the ligand-binding site, whereas *SA* is the rate of occurrence of amino acid *x* on the protein surface.

Each type of amino acid can have 40 MF-RAs according to its dihedral angles and SASA, and detailed information for the MF-RAs of the 20 amino acids is shown in Table 1.

### Protein cavity calculation and protein-ligand binding site prediction

Soga *et al*. used the Alpha Site Finder to identify and catalogue protein cavities, and all cavities were re-ranked according to the sums of the RA values of the residues involved in the cavities (PLB). The top three clusters were retained, and the top cluster was selected as the predicted ligand-binding site. Our protein cavity calculation consists of two steps. First, Ligiste-cs was employed to detect solvent grids. Ligiste-cs divides all grids projected from the protein into three groups: protein grids, surface grids and solvent grids^15^. A surface-solvent-surface event occurs when a sequence of grids starts and ends with surface grids and contains solvent grids (exceeding the minimal threshold of six grids) in the middle. Solvent grids in the surface-solvent-surface event are labelled pocket grids. Second, the depth-first search algorithm clusters all pocket grids because the web server does not provide clustering information. In this study, the clustering result should satisfy the following condition: The distance between any two pocket grids from different clusters must exceed 3.0 Å.

Before predicting the protein-ligand binding site, we defined the residues involved in the cavities: For each pocket grid cluster, the involved residue needs to have at least one non-hydrogen atom located within 4.5 Å of any grid in the cluster. All cavities were then re-ranked by their MF-PLB, which was calculated by summing the MF-RA values of all of the residues in the cavity. The cavities with the top three MF-PLB values were retained.

### Comparison measures

All cavities calculated by Ligsite-cs were re-ranked based on the MF-PLB, PLB and Ligsite-csc. Ligsite-csc re-ranks cavities according to the degree of conservation of the residues in the cavity^15^,^37^. Based on this approach, the protein-ligand binding site cavity has at least one pocket grid within 4.0 Å of any atom of the actual ligand. This method successfully predicted whether the top-scoring cavity was the actual ligand-binding site (top 1) or whether any one of the top three cavities is the actual ligand-binding site (top 3).

## Results

We evaluated the ligand-binding site prediction performances of the PLB, MF-PLB, Ligsite-csc and other methods. Two benchmark databases (set **T** and set **S**) and two evaluation criteria (top 1 and top 3) were employed. Table 2 shows a detailed comparison of the methods based on an analysis of 210 ligand-bound structures, and Table 3 shows the results of a detailed comparison of the methods based on an analysis of 48 ligand-bound and 48 ligand-unbound structures. The tables also list the prediction success rates of several other methods, namely MPK1, MPK2, Q-SiteFinder, LISE, PASS, SURFACE and SiteHound. Overall, the performance of the PLB for the analysis of 210 ligand-bound structures was found to be similar to that of Ligsite-csc, whereas the PLB achieved better performance in the prediction of the top choice from the analysis of the database of 48 ligand-bound and 48 ligand-unbound structures. The MF-PLB showed improved success rates of up to 5% (210 ligand-bound structures), 4% (48 ligand-bound structures), and 8% (48 ligand-unbound structures) for the top choice prediction compared with the PLB. When the top 3 predicted choices were used, the MF-PLB and PLB exhibited similar prediction success rates. This finding suggests that the MF-PLB can re-rank the cavities more accurately and reflect the amino acid ligand-binding site preferences better than the PLB.

MF-PLB is superior to MPK1, Q-SiteFinder, Ligsite-cs, Pass, Surface and SiteHound for both set **T** and set **S**, particularly with respect to its top 1 prediction. In general, combined ligand-binding site prediction methods show better success rates than those that utilize only geometrical information. MPK2 and LISE have higher success rates than MF-PLB for the set of 210 ligand-bound structures and the set of 48 ligand-bound structures because these two methods are meta-predictors, whereas the MF-PLB exhibited the best top-1-prediction performance in the analysis of the set of 48 ligand-unbound structures. The use of ligand-unbound structures for the accurate prediction of ligand-binding sites is more important than the use of the ligand-bound forms because the ligand coordinates in the protein structure are unknown in practical applications. A detailed comparison of the performances of Ligsite-csc, PLB and MF-PLB in the analysis of the set of 48 ligand-unbound structures is shown in Table 4.

## Discussion

**Figure 2:**
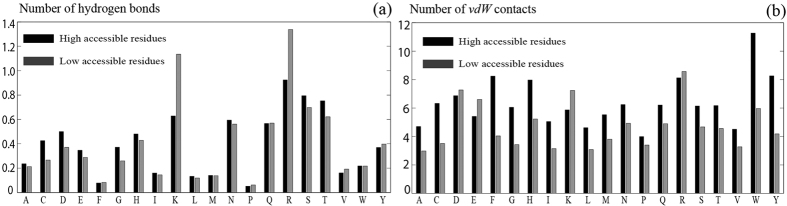
Average number of hydrogen bonds (**a**) and *vdW* contacts (**b**) formed between a ligand and a low-accessibility or a high-accessibility residue.

In this study, we show that MF-PLB exhibits good performance in ligand-binding site identification. We divided the residues into two types: low-accessibility and high-accessibility residues. Thus, it was interesting to assess the interactions between a ligand and these types of residues. Figure 2a details the average number of hydrogen bonds formed between a ligands and low-accessibility and high-accessibility residues. Approximately half of the residues establish similar numbers of hydrogen bonds in both their low- and high-accessibility states. Cys, Asp, Gly, Ser, and Thr form more hydrogen bonds in their high-accessibility states than in their low-accessibility states. The only residues that show significantly more hydrogen bonds with a ligand in their low-accessibility state compared with their high-accessibility state are the two positively charged amino acids: Lys and Arg.

We then examined the number of *vdW* contacts between a ligand and each residue involved in the ligand-binding site in both their low-accessibility and high-accessibility states (Fig. 2b). The results show that on average, high-accessibility residues form more *vdW* contacts with a ligand than low-accessibility residues with the exception of Glu and Lys. The analysis of the three amino acids with electrically charged side chains (Asp, Arg and His) showed that Asp and Arg have similar *vdW* contacts with ligands in both their high-accessibility and low-accessibility states, whereas the imidazole ring allows the high-accessibility His to establish many more *vdW* contacts with a ligand.

The average numbers of *vdW* contacts for every atom in each type of amino acid are listed in Table S5. Most atoms in the high-accessibility residues can provide more *vdW* contacts with a ligand than those in the low-accessibility residues. This finding is expected because the high-accessibility residues are more exposed on the protein surface, whereas the low-accessibility residues are relatively buried in the protein core. However, the atoms in an electrically charged side chain of a low-accessibility residue form many more *vdW* contacts with a ligand: for instance, “NZ” in low-accessibility Lys forms an average of 3.09 *vdW* contacts with a ligand, whereas “NZ” in high-accessibility Lys forms only 1.72 *vdW* contacts with a ligand. The hydrogen bonds between a ligand and high-accessibility and low-accessibility residues are shown in Fig. 2.

Set **L** consists of 169 structures and was used to evaluate the performances of different methods for small-volume ligand-binding site prediction because large-volume ligand sites can be easily detected. As shown in Table 5, almost all methods showed significantly worse performance for small-volume ligand-binding site prediction; in fact, the prediction success rates achieved with the small-volume ligand-binding site database were more than 20% lower than those achieved with the 210 ligand-bound structure database. Detailed information of the comparison can be found in Table S6. The results suggest that small-volume ligand-binding sites are relatively difficult to detect using the currently available methods. Although ConCavity shows improved performance in drug-binding site prediction^18^, it provides the fewest candidate cavities (average of 2.4 cavities) and achieves success rates of 49.1% and 67.4% for the top 1 and top 3 hits. In contrast, Surfnet supplies as many as 47.4 candidate cavities but shows the worst performance for both the top 1 and top 3 hits. LISE exhibits the highest success rate, followed by our MF-PLB index, for both benchmarks. MF-PLB performed better in small-volume ligand-binding site identification than both PLB and Ligsite-csc, with success rates that were approximately 5% higher than those of PLB for both the top 1 and top 3 hits and approximately 10% higher than that of Ligsite-csc for the top 1 hits. In conclusion, although the MF-PLB, PLB and Ligsite-csc methods show worse performance in small-volume ligand-binding site prediction, MF-PLB achieves the highest success rate among the three methods.

Another particular ligand-binding site, namely a ligand site that binds to a protein-protein interface, was taken for comparing methods prediction ability^38^. Most protein-protein interfaces are planar in shape, whereas the majority of ligand-binding sites are concave. As a result, residues in the ligand-binding sites can create more contacts with ligands. Although both protein-protein interaction and protein-ligand interaction play key roles in biological processes^39^, few studies have focused on the ligand-binding sites located at the protein-protein interface. The analysis of set **P** revealed that LISE exhibited the best performance, followed by MF-PLB and ConCavity (shown in Table 6). Two energy-based prediction methods, Q-SiteFinder and SiteHound, achieved lower success rates than the other combined methods. Detailed information of the comparison can be found in Table S7. Although various methods, such as MF-PLB, ConCavity, MPK2, exhibit good performance in the prediction of regular ligand-binding sites, the accurate identification of some particular sites, such as small-volume ligand-binding sites and ligand-binding sites on protein-protein interfaces, remains a challenging problem in the field. In the future, more physicochemical properties should be incorporated to address this problem.

**Figure 3:**
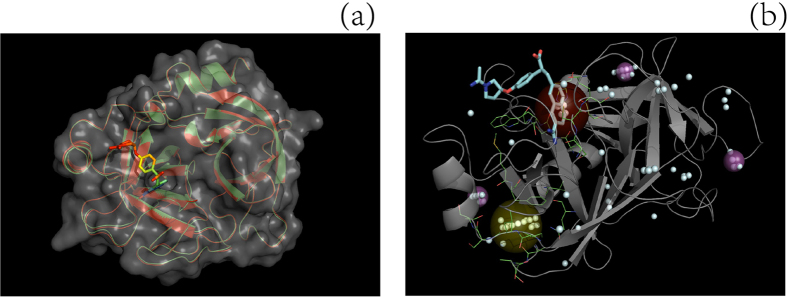
Comparison of the performance of different methods in predicting the ligand-binding site of a protein (ID: 2TGA). (**a**) illustrates how ligand coordinates and atomic connectivity information for ligand-unbound structures can be obtained from their corresponding ligand-bound proteins: A ligand-bound protein, 1MTW.pdb (red model), is aligned to the ligand-unbound protein, 2TGA.pdb (green model), using PyMOL, and the coordinates of the ligand DX9 (stick model) are then saved in the pdb file of 2TGA.pdb. The actual ligand-binding sites are the first, fourth, and fifth cavities after re-ranking by MF-PLB, PLB, and Ligsite-csc, respectively; thus, only MF-PLB successfully identifies the ligand-binding site as the top 1 hit. The MF-PLB for the actual ligand-binding site is 13.74, whereas the PLB for the actual ligand-binding site is only 11.36. The MF-PLB value of the first cavity re-ranked by the PLB (yellow ball; this cavity is also the first cavity re-ranked by Ligsite-csc) is 13.22, whereas the PLB of the cavity is 12.7. The other three cavities in the top 5 hits after re-ranking by Ligsite-csc are shown as purple balls, and all pocket grids calculated by Ligsite-cs are shown as cyan grids.

**Figure 4:**
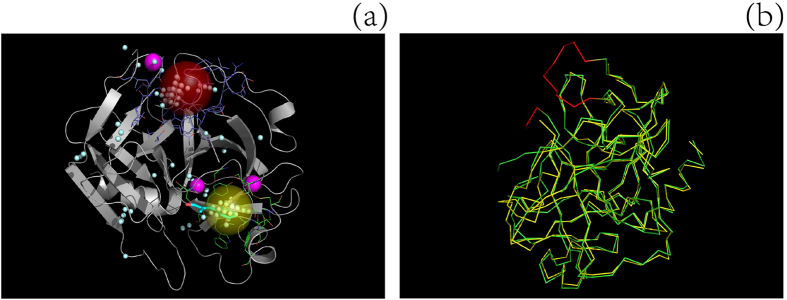
Comparison of the ligand-binding sites on a protein ((**a**) ID: 1CHG) predicted using different methods and alignment of the ligand-unbound structure with the ligand-bound structure to obtain missing Cα backbone atoms (**b**). The actual ligand-binding site (yellow ball) was the second cavity after re-ranking by the MF-PLB, whereas the first cavity (red ball) assigned by the MF-PLB is 16.63 Å away from the ligand (OAC). After examining the protein pdb file, we found that some residues were missing (red part in **b**) and that the presence of these missing residues would result in an abnormal cavity (red ball in **a**). We added these missing residues to protein 1CHG by aligning 1CHG to its ligand-bound form (3GCH) using PyMOL. The red-ball binding site was not obtained after the pocket grids were calculated by Ligsite-cs, and the first cavity assigned by the MF-PLB after the addition of the missing residues was the actual ligand-binding site. The MF-PLB values for the red-ball cavity and the yellow-ball cavity are 23.93 and 20.55, respectively. All pocket grids calculated by Ligsite-cs are shown as cyan grids.

**Figure 5:**
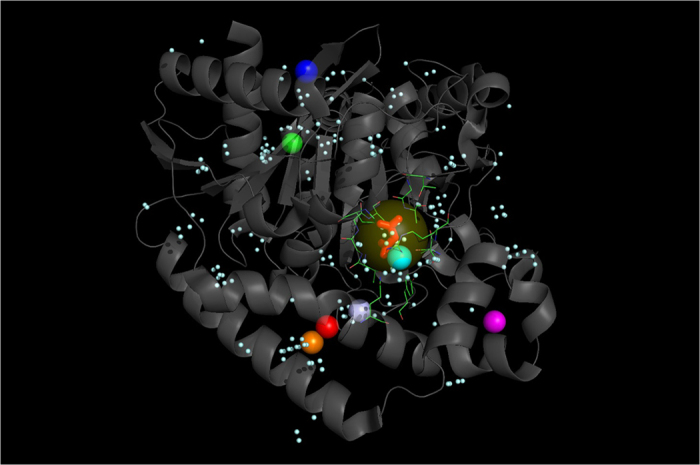
Comparison of the ligand-binding sites of a protein (ID: 1O9P) predicted using different methods. The top predictions obtained using Ligsite-csc (green ball), PLB (purple ball), MPK2 (red ball), Q-SiteFinder (orange ball), SiteHound (blue) and LISE (pink ball) are distant from the actual ligand-binding site (MLA, red stick model), and only MF-PLB (yellow ball) and ConCavity (cyan ball) were capable of correctly identifying the ligand-binding site. The MF-PLB values for the yellow-ball (residues involved: 111, 112, 113, 130, 131, 152, 155, 158, 183, 277, 356, and 359; shown in green lines) and purple-ball cavities are 18.74 and 14.58, respectively. All pocket grids calculated by Ligsite-cs are shown as cyan grids.

Three typical examples, two from set **S** and one from set **L**, are provided in Fig. 3 (id: 2tga), Fig. 4 (id: 2sil) and Fig. 5 (id: 1chg) to illustrate the procedure underlying the prediction of a ligand-binding site by the MF-PLB.

**Figure 6:**
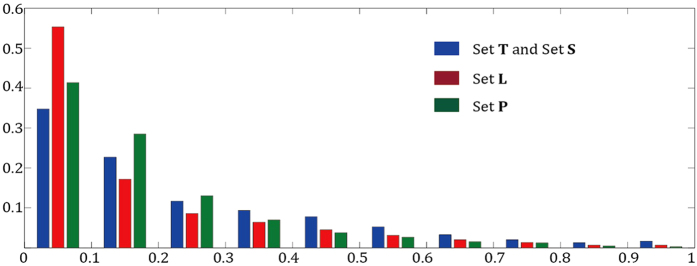
Distribution of the relative *SASA* of the residues in the ligand-binding site. The *x* axis is the range of relative *SASA* values, whereas the *y* axis is the frequency.

The MF-PLB re-ranks all of the cavities calculated by Ligsite-cs; thus, a prerequisite for an accurate prediction using the MF-PLB is that Ligsite-cs successfully finds the ligand-binding sites. The ability of Ligsite-cs to detect ligand-binding sites directly influences the prediction results of the MF-PLB approach. The MF-PLB shows lower prediction success rates with sets **L** and **P** compared with those obtained using sets **T** and **S** for the following three reasons. First, Ligsite-cs shows a lower ability to detecting ligand-binding sites in sets **L** and **P**. The success rates for the detection of ligand sites in sets **T** and **S** using Ligsite-cs are 98.1% and 97.9%, respectively, whereas the corresponding values for sets **L** and **P** are 94.6% and 85.8%. Furthermore, the MF-PLB of one cavity is calculated by summing up the MF-RA values of all residues surrounding the cavity; thus, larger cavities with more residues tend to have a higher MF-PLB. The average molecular weight of the ligands in sets **T** and **S** are 327 and 269 dalton, whereas that of the ligands in set **L** is only 126 dalton. In addition, the residues located at two particular ligand-binding sites, namely small-volume sites and protein-protein interface sites, are buried more than they are in sets **L** and **P**: the average relative *SASA* of the ligand-binding site residues in sets **L** and **P** is 16.1% and 17.6%, respectively, whereas that of the ligand site residues in sets **T** and **S** is 25.7% (shown in Fig. 6). The MF-PLB might not accurately reflect the residue preference for ligand-binding sites in sets **L** or **P** because the exposure status of the ligand-binding site residues in sets **T** and **S** are different from the typical status. The ligand sites in set **L** are buried and have a small volume, whereas the ligand-binding sites on protein-protein interfaces are planar in shape and are also buried in the protein. These reasons could explain why MF-PLB do not exhibit good performance in the analysis of sets **L** and **P**.

## Conclusion

The accurate identification of ligand-binding sites in proteins is a very important step in protein function determination and structure-based drug design. Soga *et al*. suggested that the amino acid compositions at the ligand-binding site are markedly different from those on the protein surface. These researchers created the PLB index based on the amino acid preferences for the ligand-binding site. We found that amino acids show different preferences for ligand-binding sites depending on their *SASA* and dihedral angles. Based on these findings, we successfully developed the MF-PLB index and applied it to the identification of ligand-binding sites in proteins. The obtained results showed that the MF-PLB can significantly improve the performance of the PLB for ligand-binding site prediction in both ligand-bound and ligand-unbound structures; therefore, the MF-PLB can better reflect the amino acid preferences for the ligand-binding site. The currently available methods, including the MF-PLB, show lower success rates in the prediction of small-volume ligand-binding sites and ligand-binding sites on protein-protein interfaces, but the MF-PLB still exhibits the best performance among the MF-PLB, PLB, and Ligsite-csc methods. Additionally, the performance of the MF-PLB was better than those of the other methods, with the exception of LISE. The calculation of the MF-PLB is simple, and clearly, this index could be a useful tool for ligand-binding site prediction and other aspects of drug discovery.

## Additional Information

**How to cite this article**: Cao, C. and Xu, S. Improving the performance of the PLB index for ligand-binding site prediction using dihedral angles and the solvent-accessible surface area. *Sci. Rep.* **6**, 33232; doi: 10.1038/srep33232 (2016).

## Supplementary Material

Supplementary Information

## Figures and Tables

**Table 1:** MF-RA for 20 amino acids.

RES	1^1^	2	3	4	5	6	7	8	9	10
A^2^	1.32	1.33	1.3	1.87	1.02	1.93	1.62	1.74	1.34	0.91
a^2^	0.53	0.58	0.55	0.85	0.68	0.88	0.78	0.82	0.71	0.61
C	1.78	2.28	3.98	2.56	2.2	4.42	2.74	2.9	3.39	2.76
c	0.65	0.63	0.81	0.86	0.61	0.71	0.34	0.69	0.65	0.71
D	1.46	1.29	0.83	0.76	0.8	0.61	0.42	0.46	0.62	0.53
d	3.26	2.48	3.61	2.65	1.68	2.36	1.58	2.27	1.49	1.73
E	0.5	0.49	0.83	0.56	0.74	0.88	0.74	0.54	0.53	0.34
e	1.96	2.21	2.61	2.87	2.95	2.13	2.36	1.72	1.62	1.37
F	1.43	1.84	1.65	1.67	2.03	1.95	2.86	1.75	1.69	2.49
f	0.68	0.83	0.92	0.89	0.87	0.88	0.95	0.57	0.8	0.85
G	2.12	2.05	2.31	3.25	2.24	1.62	1.85	1.54	3.27	1.6
g	0.9	0.83	0.98	0.99	1.27	1.11	1.04	1.04	1.76	0.92
H	1.47	1.56	1.08	1.17	1.58	1.16	1.98	1.29	0.93	1.45
h	2.14	2.49	2.37	2.48	1.77	2.68	3.31	2.43	1.49	1.87
I	1.41	1.43	1.69	1.34	1.61	2.52	1.33	2.21	2.16	2.07
i	0.61	0.66	0.51	0.58	0.6	0.61	0.38	0.7	0.89	0.65
K	0.51	0.39	0.33	0.56	0.47	0.39	0.43	0.44	0.39	0.43
k	3.46	3.39	2.35	5.73	3.83	2.61	3.02	3.96	3.61	3.92
L	1.46	1.63	1.46	1.41	1.23	1.88	1.6	1.53	1.48	1.2
l	0.65	0.93	0.55	0.61	0.54	0.46	0.49	0.48	0.59	0.45
M	1.77	2.45	1.94	2.15	1.68	2.08	2.26	2.27	2.24	1.48
m	0.74	1.36	0.83	1.15	0.78	0.69	0.89	0.98	0.83	0.61
N	1.01	1.27	0.76	0.87	1.16	0.41	0.73	0.78	0.91	0.75
n	1.71	1.92	1.63	2.11	1.57	0.99	1.48	1.31	1.66	1.48
P	1.5^3^	0.42	1.5	0.78	0.54	1.5	0.51	0.62	0.48	0.78
p	1.5	0.44	1.5	1.16	0.68	1.5	0.55	0.66	0.27	0.8
Q	0.53	0.57	0.46	0.58	0.85	0.46	0.75	0.68	0.5	0.63
q	1.45	1.98	1.17	1.96	1.88	1.09	1.56	1.47	1.47	1.7
R	0.71	0.66	0.78	0.68	0.97	0.86	1.08	0.93	0.87	1.03
r	2.88	2.71	3.05	3.74	2.59	2.39	2.75	2.43	2.52	2.94
S	0.79	0.84	1.47	0.92	1.31	1.11	1.51	1.05	0.94	0.99
s	1.13	0.84	1.53	1.36	1.13	1.08	1.24	1.07	0.95	1.2
T	0.78	0.68	0.68	0.54	0.96	0.9	1.46	1.03	0.68	1.42
t	0.89	1.03	0.73	1.05	1.12	0.82	1.13	1.03	0.9	1.4
V	1.06	1.05	0.9	1.14	1.47	1.92	1.47	1.8	1.1	2.03
v	0.48	0.53	0.37	0.57	0.67	0.6	0.57	0.67	0.58	0.67
W	1.77	1.9	1.71	2.03	2.59	1.69	3.89	3.8	2.75	3.12
w	1.6	1.28	1.33	1.6	1.44	0.98	1.25	1.2	1.4	1.2
Y	1.12	1.17	1.26	1.33	1.97	1.34	2.13	1.66	1.09	2.03
y	1.2	1.07	1.43	1.5	1.65	1.28	1.59	1.23	0.96	1.14
RES	11	12	13	14	15	16	17	18	19	20^4^
A	0.82	1.43	0.77	1.58	1	0.81	2.47	0.32	0.91	1.54
a	0.63	0.84	0.45	0.86	0.75	1.25	1.01	0.56	0.72	0.89
C	3.08	2.01	3.73	2.96	3.3	1.5	4.62	3.11	2.54	3.07
c	0.72	0.96	0.75	1.29	0.58	1.5	0.63	2.82	0.71	1.35
D	0.76	0.58	0.52	0.57	0.31	2.7	1.12	3.55	0.83	0.7
d	2.16	1.08	1.92	1.87	0.6	0.91	2.3	2.62	1.88	1.41
E	0.45	0.57	0.31	2.11	0.35	1.5	1.33	0.63	0.44	0.4
e	1.72	2.6	1.69	4.78	0.93	1.5	2.64	1.01	1.96	0.77
F	2.94	1.73	2.75	2.51	2.9	3.38	3.45	1.56	1.44	1.85
f	0.87	0.83	1.01	1.31	1.52	1.87	1.03	1.3	0.74	0.89
G	1.88	3.96	2.79	1.26	1.45	0.96	1.33	0.34	1.62	1.51
g	1.07	2.37	1.32	0.69	1.29	0.98	0.73	0.49	0.95	1.48
H	1.59	0.97	1.45	2.74	3.65	1.5	2.73	6.98	1.27	1.22
h	1.75	1.19	2.18	2.65	1.72	1.5	3.05	3.33	1.98	3.64
I	2.23	1.19	1.86	1.07	0.68	1.5	5.84	1.2	1.02	0.9
i	0.65	0.6	0.64	0.39	0.24	1.5	1.87	0.94	0.65	0.55
K	0.54	0.32	0.54	0.42	0.44	1.5	1.18	0.38	0.38	0.81
k	4.73	3.27	5.08	8.1	3.5	1.5	9.31	2.11	3.11	3.6
L	1.26	1.36	1.42	0.3	0.72	4.16	2.28	1.43	0.89	0.76
l	0.45	0.68	0.53	0.16	0.41	2.5	0.6	0.49	0.57	0.52
M	2.02	1.59	2.38	1.94	0.68	1.5	2.2	1.11	0.6	0.89
m	0.78	0.79	0.91	1.45	0.67	1.5	0.63	0.7	0.81	0.36
N	1.01	1.28	1	0.3	0.54	1.5	1.28	0.56	1.08	0.5
n	1.63	2.74	1.99	0.68	0.87	1.5	1.08	1.21	1.45	0.63
P	0.54	1.5	0.51	1.5	0.52	1.5	1.5	1.5	0.47	0.33
p	0.81	1.5	1.15	1.5	0.43	1.5	1.5	1.5	0.87	5.51
Q	0.66	0.48	0.48	1.1	0.53	1.5	0.7	0.75	0.52	0.15
q	1.58	1.44	1.5	1.76	1.23	1.5	1.4	1.06	1.4	0.41
R	1.07	0.78	0.77	1.26	0.38	2.25	1.18	0.61	0.7	0.8
r	2.92	2.27	3.37	5.06	2.67	3.75	1.68	1.13	2.62	1.89
S	0.87	1.27	1.23	0.61	1.15	3.49	1.79	1.07	0.95	0.93
s	1.14	1.33	1.33	0.82	0.9	1.67	1.38	0.79	1.09	0.58
T	1.16	0.79	1.69	0.94	1.01	1.02	2.01	2.03	0.77	0.54
t	1.05	1.1	1.63	0.68	0.63	1.56	0.82	0.65	0.82	0.4
V	2.1	0.84	1.39	0.79	1.3	1.5	4.87	1.11	0.61	0.85
v	0.73	0.56	0.55	0.52	0.26	1.5	1.34	0.66	0.47	0.53
W	2.72	1.92	2.59	3.9	2.04	1.5	6.06	1.95	2.41	1.11
w	1.19	1.17	1.32	4.49	2.67	1.5	1.66	1.21	1.35	1.1
Y	2.18	1.92	1.89	2.59	2.32	1.35	2.4	2.31	1.64	0.85
y	1.11	1.33	1.45	2.09	2.05	0.62	1.05	1.13	1.35	0.69

^1^Region in the Ramachandran plot.

^2^Capital letters indicate high-accessibility amino acids (relative SASA > 13.1%), whereas lowercase letters indicate low-accessibility amino acids (relativeSASA ≤ 13.1%).

^3^The backbone conformation is considered rare if fewer than 20 residues are located in the region, and the MF-RA value of rare-conformation amino acids is 1.5 based on Petock *et al*., who reported that these residues are likely to be located at the functional site^35^.

^4^DISICL divided the Ramachandran plot into 19 regions, and the rest of the area in the Ramachandran plot was considered the 20^th^ region^36^.

**Table 2:** Comparison of the performance of different methods in the analysis of 210 ligand-bound structures.

Method	Top 1 (%)	Top 3 (%)
MF-PLB	80	93
PLB	75	92
MPK2^1^	81	95
MPK1^2^	75	93
Q-SiteFinder^2^	70	90
LISE^3^	83	94
Ligsite-csc^4^	75	—
Ligsite-cs^4^	70	86
PASS^4^	51	80
SURFACE^4^	42	57
SiteHound	65	81

^1^Data from^20^.

^2^Data from^21^.

^3^Data from^19^.

^4^Data from^15^.

**Table 3:** Comparison of the performance of different methods in the analysis of 48 ligand-bound/unbound structures.

Method	Bound Protein		Unbound Protein	
	Top 1 (%)	Top 3 (%)	Top 1 (%)	Top 3 (%)
MF-PLB	85	96	**83**	**92**
PLB	81	94	**75**	**90**
MPK2^1^	85	96	80	94
MPK1^2^	83	96	75	90
Q-SiteFinder^2^	75	90	52	75
LISE^3^	92	96	81	92
Ligsite-csc^4^	79	—	71	—
Ligsite-cs^4^	81	92	71	85
PASS^4^	63	81	60	71
SURFNET^4^	54	78	52	75
SiteHound	71	90	63	81

^1^Data from^20^.

^2^Data from^21^.

^3^Data from^19^.

^4^Data from^15^.

**Table 4:** Comparison of the performances of Ligsite-csc, PLB and MF-PLB in the analysis of ligand-unbound structures.

Bound Prot	Unbound Prot	Hit^1^			D_near_(Å)^2^
		Ligsite-csc	MF-PLB	PLB	
1bid	3tms	1	1	1	3.4
1cdo	8adh	1	1	1	0.8
1dwd	1hxf	1	1	1	1.7
1fbp	2fbp	1	1	1	0.5
1gca	1gcg	1	1	1	0.8
1hew	1hel	1	1	1	1.8
1hyt	1npc	1	1	1	1.2
1inc	1esa	1	3	5	2.9
1rbp	1brq	1	1	1	0.9
1rob	8rat	1	1	1	0.9
1stp	1swb	1	1	1	0.6
1ulb	1ula	—	—	—	—
2ifb	1ifb	1	1	1	2.2
3ptb	3ptn	2	1	2	1.1
2ypi	1ypi	—	1	1	3.0
4dfr	5dfr	1	1	1	1.9
4phv	3phv	1	6	7	2.7
5cna	2ctv	11	3	3	1.0
7cpa	5cpa	1	1	1	1.0
1a6w	1a6u	3	5	3	0.5
1acj	1qif	—	1	1	3.5.
1apu	3app	—	1	1	1.2
1blh	1djb	1	1	1	0.7
1byb	1bya	1	1	1	2.5
1hfc	1cge	1	1	1	0.7
1ida	1hsi	1	1	2	3.4
1igj	1a4j	4	8	7	0.8
1imb	1ime	1	1	1	1.7
1ivd	1nna	1	1	1	1.4
1mrg	1ahc	1	1	1	1.9
1mtw	2tga	5	1	4	2.8
1okm	4ca2	1		1	1
1pdz	1pdy	1	1	1	2.6
1phd	1phc	1	1	1	0.7
1pso	1psn	1	1	1	0.8
1qpe	3lck	1	1	1	1.5
1rne	1bbs	1	1	1	1.0
1snc	1stn	1	1	1	1.5
1srf	1pts	1	1	1	1.5
2ctc	2ctb	1	1	1	0.6
2h4n	2cba	2	1	1	1.0
2pk4	1krn	2	1	1	0.7
2sim	2sil	2	2	2	0.7
2tmn	1l3f	—	1	1	2.1
3gch	1chg	1	2	3	2.2
3mth	6ins	1	1	1	3.8
5p2p	3p2p	1	1	1	1.3
6rsa	7rat	4	1	3	0.9

^1^Best ranking of ligand-binding site.

^2^Distance between the ligand and the central pocket grid of the ligand-binding site.

**Table 5:** Comparison of the performance of different methods in the analysis of 169 small-volume ligand-binding site structures.

Method	MF-PLB	PLB	Ligsite-csc
Top 1 (%)	58.6	52.6	47.9
Top 3 (%)	76.9	71.5	69.8
**Method**	**ConCavity**	**Surfnet**	**Q-SiteFinder**
Top 1 (%)	49.1	21.9	31.9
Top 3 (%)	67.4	38.5	59.7
**Method**	**MPK2**	**LISE**	**Site-Hound**
Top 1 (%)	44.3	62.1	30.2
Top 3 (%)	66.2	79.9	55.0

**Table 6:** Comparison of the performance of different methods in the analysis of 149 structures of ligand-binding sites on protein-protein interface.

Method	MF-PLB	PLB	Ligsite-csc
Top 1 (%)	57.7	53.0	51.6
Top 3 (%)	71.8	69.1	66.4
**Method**	**ConCavity**	**Surfnet**	**Q-SiteFinder**
Top 1 (%)	55.0	21.5	48.3
Top 3 (%)	73.2	32.9	65.7
**Method**	**MPK2**	**LISE**	**Site-Hound**
Top 1 (%)	48.9	63.8	43.0
Top 3 (%)	67.1	81.9	65.1
