# Supplementary material for: Improving the performance of the PLB index for ligand-binding site prediction using dihedral angles and the solvent-accessible surface area
Source: Sci Rep. 2016 Sep 13;6:33232. doi: 10.1038/srep33232 (PMC5020399; doi:10.1038/srep33232)
Supplement: Supplementary Information [file srep33232-s1.pdf]

Supplementary Information

Improving the performance of the PLB index for ligand-binding site prediction using dihedral angles and the solvent-accessible surface area

Chen Cao[1,2*], Shutan Xu[3]

[1]College of Computer Science and Technology, Jilin University, Changchun, Jilin, China

[2]Key Laboratory of Symbol Computation and Knowledge Engineering of the Ministry of Education, Jilin University, Changchun, Jilin, China

[3]Department of Biochemistry and Molecular Biology, Institute of Bioinformatics, University of Georgia, Athens, GA, USA

*Corresponding author: Chen Cao (email: caochen13@mails.jlu.edu.cn)

Supplementary Tables S1-S7

```
import requests
import re
import time
import os

def getpdb(pdbid,chain):
    data={
    "pdbid":pdbid,
    "chain":chain,
    "email":'',
    "number":3,
    }
    session=requests.session()
    html=session.post('http://projects.biotec.tu-dresden.de/mpcgi/metapocket.cgi',data=data).text
    url=re.findall('URL=(http.*?.html)',html)[0]
    count=0
      while True:
        html=session.get(url,timeout=200).text
        urls=re.findall('href="(http.*?pdb)"',html.replace(' ',''))
        if urls==[]:
            time.sleep(1)
            if count==100:
                return False
            count+=1
            continue
        break
    for url in urls:
        try:
            content=requests.get(url,timeout=200).content
            with open('result/'+url.split('/')[-1],'wb') as pdbfile:
                pdbfile.write(content)
        except:
            print(url,'--failed')
    return True

def main():
    try:
        os.mkdir('result')
    except:
        pass
    for line in open('list.txt','r'):
        line=line.replace('\n','')
        lists=line.split(' ')
        try:
            statue=getpdb(lists[0],lists[1])
        except:
            print(line,'--failed')
        if(statue==False):
            print(line,'--No result')
            continue
        print(line,'--ok')
main()
```

Table S1. Python script for setting parameters and downloading prediction results from the MPK2 web server automatically. The Python script automatically downloads the ligand-binding site prediction results from various methods (including

Surfnet, ConCavity, MPK2, and Q-SiteFinder) and the file of pocket grids for the protein calculated by Ligsite-cs.

```
import requests
import threading
import re
import os

class GetPdb(threading.Thread):
	def __init__(self,pdbid,chain):
		super(GetPdb,self).__init__()
		self.filename=pdbid+'-'+chain+'.pdb'
		self.data={
		"pdbid":pdbid,
		# "chain":' ',
		"rerank":'on',
		"space":1.0,
		"number":5,
		"radius":5.0,
		"submit":'Find pockets'
		}
	def run(self):
		html=requests.post('http://projects.biotec.tu-dresden.de/cgi-bin/pocketfinder.cgi',data=self.data).text
		try:
			url=re.findall('href="(.*?\.pdb)"',html)[0]
		except:
			pass
		content=requests.get('http://projects.biotec.tu-dresden.de'+url).content
		with open('result/'+self.filename,'wb') as filew:
			filew.write(content)

def main():
	try:
		os.mkdir('result')
	except:
		pass
	for line in open('list.txt','r'):
		line=line.replace('\n','')
		lists=line.split(' ')
		try:
			work=GetPdb(lists[0],lists[1])
			work.run()
		except:
			print(line,'--failed')
		print(line,'--ok')
main()
```

Table S2. Python script for setting parameters and downloading prediction results from the Ligsite-csc web server automatically. The Python script automatically downloads the ligand-binding site prediction results obtained using Ligsite-csc.

```
import requests
from bs4 import BeautifulSoup
import os
import time

def upload(filename):
	data={
	'SITE_NAME':'SITEHOUND',
	'pdb_id':'',
	'probe':'CMET',
	'algorithm':'a',
	'filename':filename,
	}
	session=requests.session()
	files={'file': open('pdb_dir/%s'%filename, 'rb')}
	html=session.post('http://scbx.mssm.edu/sitehound/sitehound-web/cgi/wait1.cgi',data=data,files=files).text
	inputs=BeautifulSoup(html,'html.parser').find_all('input')
	data={}
	for item in inputs:
		data[item.get('name')]=item.get('value')
	html=session.post('http://scbx.mssm.edu/sitehound/sitehound- web/cgi/protein_processing.cgi',data=data).text
	inputs=BeautifulSoup(html,'html.parser').find_all('input')
	data={}
	for item in inputs:
		data[item.get('name')]=item.get('value')
	html=session.post('http://scbx.mssm.edu/sitehound/sitehound-web/cgi/wait3.cgi',data=data).text
	inputs=BeautifulSoup(html,'html.parser').find_all('input')
	data={}
	for item in inputs:
		data[item.get('name')]=item.get('value')
	html=session.post('http://scbx.mssm.edu/sitehound/sitehound-web/cgi/sitehound.cgi',data=data).text
	url=BeautifulSoup(html,'html.parser').find('div',id='main').find('a').get('href')
	while True:
		html=session.get(url).text
		if 'Cluster Data' in html:
			try:
				os.mkdir('result')
			except:
				pass
			f=open('result/%s.txt'%filename,'w',encoding='utf-8')
			result=BeautifulSoup(html,'html.parser').find('td',{'align':'center'}).find('table').find_all('tr')
			for tr in result:
				for td in tr.find_all('td'):
					f.write(td.get_text().replace('\r','').replace('\n','')+'\t')
				f.write('\r\n')
			f.close()
			print(filename,'ok')
			return
		print(filename,'sleep')
		time.sleep(15)

def main():
	for filename in os.listdir(' pdb_dir '):
		try:
			upload(filename)
		except:
			failed=open('failed.txt','a',encoding='utf-8')
```

```
            failed.write(filename+'\n')
            failed.close()

main()
```

Table S3. Python script for uploading proteins, setting parameters and downloading prediction results from the SiteHound web server automatically.

Region	ϕ_{min}	ϕ_{max}	φ_{min}	φ_{max}
01	-180	-135	136	180
02	-135	-105	150	180
03	-175	-135	95	136
04	-135	-87	95	150
05	-87	-30	95	180
06	-170	-113	50	95
07	-113	-60	50	95
08	-150	-67	8	40
09	-150	-107	-32	8
10	-107	-40	-12	8
11	-107	-40	-32	-12
12	-165	-95	-70	-32
13	-95	-40	-70	-32
14	-180	-135	-180	-160
15	-100	-60	-180	-150
16	60	120	150	180
17	38	140	-25	75
18	55	95	-100	-40
19	35	100	-180	-110

Table S4. Detailed information for the twenty regions defined by DISICL. Angles are shown in degrees, and the rest of the Ramachandran plot constitutes the 20th region.

AA name	At Name	High-accessibility	Low-accessibility
A	N	0.80	0.58
A	CA	0.70	0.43
A	C	0.44	0.33
A	O	0.83	0.50
A	CB	1.93	1.14
C	N	0.55	0.43
C	CA	0.59	0.45
C	C	0.36	0.42
C	O	0.77	0.71
C	CB	1.68	0.49
C	SG	2.38	1.01
D	N	0.47	0.14
D	CA	0.43	0.20
D	C	0.27	0.15
D	O	0.43	0.14
D	CB	0.55	0.20
D	CG	1.19	1.43
D	OD1	1.69	2.22
D	OD2	1.84	2.79
E	N	0.24	0.09
E	CA	0.27	0.17
E	C	0.25	0.16
E	O	0.61	0.15
E	CB	0.28	0.13
E	CG	0.46	0.22
E	CD	0.86	1.29
E	OE1	1.16	2.16
E	OE2	1.28	2.24
F	N	0.23	0.22

F	CA	0.23	0.18
F	C	0.17	0.18
F	O	0.29	0.31
F	CB	0.61	0.28
F	CG	0.65	0.20
F	CD1	1.00	0.34
F	CD2	0.97	0.38
F	CE1	1.36	0.59
F	CE2	1.26	0.61
F	CZ	1.47	0.75
G	N	1.56	0.93
G	CA	2.28	1.27
G	C	1.10	0.70
G	O	1.11	0.53
H	N	0.28	0.13
H	CA	0.27	0.14
H	C	0.23	0.12
H	O	0.38	0.22
H	CB	0.62	0.23
H	CG	0.61	0.22
H	ND1	1.03	0.50
H	CD2	1.11	0.81
H	CE1	1.61	1.17
H	NE2	1.83	1.69
I	N	0.40	0.33
I	CA	0.25	0.21
I	C	0.14	0.22
I	O	0.39	0.49
I	CB	0.41	0.21
I	CG1	0.78	0.36
I	CG2	1.09	0.62
I	CD1	1.59	0.71

K	N	0.45	0.94
K	CA	0.40	0.35
K	C	0.18	0.22
K	O	0.30	0.09
K	CB	0.45	0.45
K	CG	0.58	0.27
K	CD	0.62	0.33
K	CE	1.17	1.40
K	NZ	1.72	3.19
L	N	0.37	0.35
L	CA	0.24	0.21
L	C	0.24	0.24
L	O	0.48	0.54
L	CB	0.47	0.31
L	CG	0.34	0.17
L	CD1	1.35	0.64
L	CD2	1.13	0.62
M	N	0.42	0.41
M	CA	0.33	0.26
M	C	0.24	0.19
M	O	0.51	0.45
M	CB	0.71	0.39
M	CG	0.79	0.50
M	SD	1.11	0.72
M	CE	1.42	0.89
N	N	0.46	0.23
N	CA	0.36	0.21
N	C	0.28	0.16
N	O	0.48	0.22
N	CB	0.67	0.31
N	CG	0.90	0.81
N	OD1	1.32	1.25

N	ND2	1.77	1.74
P	N	0.20	0.18
P	CA	0.34	0.28
P	C	0.27	0.37
P	O	0.59	0.59
P	CB	0.62	0.56
P	CG	0.98	0.69
P	CD	0.99	0.73
Q	N	0.40	0.17
Q	CA	0.36	0.14
Q	C	0.26	0.12
Q	O	0.46	0.19
Q	CB	0.54	0.19
Q	CG	0.67	0.28
Q	CD	0.88	0.80
Q	OE1	1.19	1.37
Q	NE2	1.45	1.64
R	N	0.39	0.13
R	CA	0.28	0.11
R	C	0.18	0.08
R	O	0.28	0.13
R	CB	0.46	0.14
R	CG	0.52	0.18
R	CD	0.79	0.48
R	NE	1.09	0.70
R	CZ	1.25	1.69
R	NH1	1.22	2.21
R	NH2	1.66	2.72
S	N	0.88	0.61
S	CA	0.73	0.55
S	C	0.41	0.37
S	O	0.54	0.42

S	CB	1.45	0.98
S	OG	2.13	1.74
T	N	0.72	0.63
T	CA	0.56	0.45
T	C	0.37	0.30
T	O	0.57	0.39
T	CB	0.99	0.69
T	OG1	1.87	1.48
T	CG2	1.09	0.62
V	N	0.42	0.57
V	CA	0.27	0.27
V	C	0.20	0.27
V	O	0.51	0.59
V	CB	0.47	0.26
V	CG1	1.23	0.61
V	CG2	1.41	0.70
W	N	0.20	0.16
W	CA	0.24	0.13
W	C	0.22	0.12
W	O	0.40	0.19
W	CB	0.58	0.20
W	CG	0.65	0.26
W	CD1	0.94	0.55
W	CD2	0.90	0.35
W	NE1	1.37	0.94
W	CE2	1.18	0.66
W	CE3	1.02	0.35
W	CZ2	1.32	0.89
W	CZ3	1.09	0.48
W	CH2	1.16	0.69
Y	N	0.25	0.12
Y	CA	0.28	0.14

Y	C	0.19	0.11
Y	O	0.32	0.16
Y	CB	0.60	0.20
Y	CG	0.61	0.16
Y	CD1	0.92	0.30
Y	CD2	0.90	0.28
Y	CE1	1.13	0.58
Y	CE2	1.10	0.61
Y	CZ	1.09	0.72
Y	OH	1.87	1.80

Table S5. Average number of *vdW* contacts between ligands and high-accessibility or low-accessibility residues.

PDB_Chain	Ligand	MF-PLB	Ligsite-csc	MPK2	ConCavity	Q-SiteFinder	Surfnet	LISE	PLB	SiteHound
1cb0_A	ADE	1	1	1	1	1	44	1	1	N
1cea_A	ACA	N	N	5	2	1	4	N	N	2
1chm_A	CMS	1	1	3	2	2	4	1	1	1
1esd_A	VXA	1	1	1	1	N	N	1	1	1
1fur_A	MLT	7	12	N	5	N	N	4	12	N
1gxu_A	2HP	1	1	6	3	5	7	N	1	N
1izc_A	PYR	1	1	N	1	N	N	2	1	8
1jlt_A	MRD	1	1	1	1	1	1	2	1	1
1lo6_A	BEN	1	1	1	1	1	N	1	2	2
1lpd_A	ADE	1	1	1	1	1	N	1	1	1
1m3u_A	KPL	1	1	1	1	1	1	2	1	1
1n2m_A	MRD	6	6	N	N	6	7	4	5	5
1nki_A	PPF	1	4	5	2	N	2	1	2	3
1o4t_A	OXL	1	1	1	1	1	1	1	1	4
1o6e_A	ISP	2	2	1	1	3	43	2	2	N
1o9p_A	MLA	1	6	3	1	3	N	2	3	N
1oi0_A	144	4	4	2	1	5	2	1	5	3
1otj_A	TAU	3	3	2	N	2	N	1	2	N
1p6o_A	HPY	1	1	3	N	4	7	1	1	9
1pot_A	SPD	2	3	3	N	5	N	1	2	3
1r9l_A	BET	1	3	2	2	5	1	1	1	2
1rcd_A	BET	4	6	5	N	7	7	7	9	6
1rd5_A	MLA	3	2	2	N	3	N	2	2	9
1ryo_A	OXL	7	26	N	N	N	N	N	7	N
1s7f_A	MLA	2	1	N	N	N	N	8	2	N
1sgj_A	OAA	1	1	1	1	1	N	1	1	2
1tkj_A	MED	1	1	1	1	1	1	1	1	1
1tt8_A	PHB	1	2	1	1	2	5	5	2	4

1w61_A	PYC	2	10	N	N	7	16	4	2	N
1w6f_A	ISZ	1	1	1	1	1	N	1	1	2
1wzu_A	MLT	1	1	1	1	N	N	1	1	4
1xff_A	ACT	1	1	1	1	1	4	1	1	1
1zei_A	CRS	5	5	2	2	3	2	6	5	1
1zuw_A	DGL	1	2	1	2	1	3	1	1	6
2a1x_A	AKG	1	1	1	1	1	N	1	1	1
2ayd_A	SIN	11	8	N	N	6	5	2	6	5
2b4l_A	BET	1	1	3	2	2	3	1	1	1
2cks_A	BEN	1	1	1	1	3	2	2	1	1
2cw6_A	3HG	1	1	1	1	1	1	1	1	1
2dt9_B	ACT	8	2	2	N	1	5	5	4	3
2f8a_A	MLA	15	13	N	N	N	N	2	15	N
2g3f_A	IZC	2	3	3	N	3	N	1	2	N
2gll_A	BEN	17	14	6	N	6	8	N	17	5
2gm8_A	HMH	1	2	5	1	3	N	1	1	4
2gso_A	VO4	1	1	1	1	N	N	2	1	N
2gzm_A	DGL	1	5	3	2	1	N	1	1	5
2h8g_A	ADE	1	1	1	1	12	N	2	1	1
2hfu_A	MEV	1	1	1	1	N	N	2	1	6
2hzl_A	PYR	2	3	5	N	10	14	4	3	N
2ifc_A	OAA	1	1	1	1	1	1	1	1	2
2iwz_A	6NA	3	4	N	N	1	23	3	3	9
2ix4_A	6NA	5	7	N	N	1	N	4	6	N
2j13_A	CAC	2	1	1	1	1	1	2	2	2
2jfq_A	DGL	1	1	1	1	2	N	1	1	N
2oq5_A	BEN	2	2	1	1	1	N	2	2	1
2p18_A	SPD	3	5	7	N	3	6	1	3	1
2p3x_A	C2O	2	35	N	N	N	N	1	2	N
2pka_B	BEN	3	3	6	N	4	N	2	3	2
2pqj_A	ADE	1	1	1	1	1	1	1	1	1
2qfy_A	AKG	1	5	2	1	7	1	1	4	1

2qhs_A	OCA	1	1	1	1	2	1	1	1	1
2qrl_A	OGA	1	1	1	1	2	N	1	1	7
2ra6_A	ETY	2	2	2	1	1	2	1	2	1
2rin_A	ACH	1	2	2	3	2	14	1	1	2
2v3u_A	DSN	2	1	1	2	2	1	2	2	N
2v62_A	SIN	22	10	N	N	18	N	9	22	N
2veq_A	CAC	9	10	N	N	N	7	6	7	N
2vkl_A	MLT	1	1	1	1	1	1	1	1	1
2vw8_A	CAC	1	1	1	1	1	1	1	1	N
2wl9_B	MBD	1	1	1	1	N	26	1	1	2
2wmm_A	MLT	5	12	7	N	N	3	9	8	8
2xrh_A	NIO	1	1	1	1	1	N	4	1	1
2xz9_A	PYR	1	1	N	N	19	N	1	1	2
2yzo_A	MLT	1	1	1	1	1	N	1	1	1
2zzv_A	LAC	3	6	N	N	12	17	1	4	N
3a1i_A	UNU	1	2	5	N	3	N	N	1	5
3aal_A	CAC	1	1	1	1	2	N	1	1	1
3au7_A	AG2	32	45	N	N	2	N	1	43	1
3b7o_A	MLT	1	3	2	2	2	N	1	1	2
3bf8_A	MLA	1	1	2	N	2	N	1	1	8
3bsf_A	ADE	1	1	1	1	1	1	2	1	1
3by8_A	MLT	1	2	5	2	4	8	1	1	6
3ct4_A	2HA	1	8	6	N	N	N	2	4	2
3dg6_A	MUC	1	2	5	2	6	8	1	3	N
3dmo_A	MRD	1	1	1	1	1	1	2	1	1
3dr3_A	MLT	6	8	N	N	N	13	3	6	3
3erp_A	CAC	24	6	2	N	5	2	1	23	3
3fpc_A	CAC	2	33	N	N	N	9	1	22	1
3gf2_A	SAL	19	13	5	N	3	N	1	19	N
3gn9_A	MLT	3	4	3	2	2	2	2	4	2
3h78_A	BE2	N	N	1	1	1	N	6	N	N
3ktm_A	BU4	N	N	8	N	3	N	N	N	N

3lqv_A	ADE	1	1	1	3	1	1	1	1	2
3n5f_B	CAC	1	1	1	1	1	39	1	1	1
3nnf_A	AKG	2	1	2	1	3	6	1	1	1
3obz_A	AKG	N	N	4	N	2	N	1	N	1
3og9_A	MLT	1	1	1	1	1	1	1	1	9
3opt_A	AKG	1	1	1	1	1	N	1	1	1
3pb6_X	CAC	1	1	1	1	N	N	1	3	1
3plx_B	PEG	3	4	4	N	5	2	4	5	7
3pua_A	OGA	4	4	3	N	1	2	1	4	2
3q12_A	PAF	1	1	1	1	2	N	1	1	1
3r2j_A	NIO	1	1	1	1	1	2	1	1	1
3r6u_A	CHT	1	1	1	1	5	1	1	1	1
3r9r_A	MRD	1	1	1	1	1	1	1	1	2
3r9t_A	BEZ	1	1	1	1	N	1	N	1	N
3rys_A	ADE	1	2	2	3	2	N	1	1	N
3s99_A	ADE	3	5	5	1	2	3	1	3	6
3t7y_A	SIN	5	2	5	N	3	21	10	4	2
3tht_A	AKG	2	2	8	2	4	4	1	2	3
3tmg_A	BET	4	2	2	2	1	4	1	3	6
3vsj_B	2XP	1	1	1	1	2	1	1	1	1
3w36_A	VO4	1	5	N	4	N	N	1	4	N
3war_A	NIO	2	2	4	N	1	2	1	2	1
3wwx_A	DIA	1	1	1	N	1	1	2	1	1
3zfp_A	SGM	8	8	N	N	N	7	N	5	9
3zha_A	SIN	7	3	2	1	8	2	N	4	5
3zmd_A	SAL	16	10	5	N	N	3	3	16	N
3zs3_A	SIN	1	1	1	1	1	1	1	1	2
3zxf_A	ACT	2	5	4	N	7	2	6	4	4
4a9v_A	FEO	5	3	3	3	2	13	1	5	2
4buh_A	PEG	22	16	4	3	N	2	2	22	N
4bxf_A	AKG	N	N	8	2	4	N	1	N	3
4c0z_A	SPD	6	13	N	N	N	14	10	4	10

4cck_A	OGA	1	1	2	N	3	1	1	1	1
4ccw_A	VKC	1	1	N	1	5	N	1	1	N
4dbh_A	OXL	1	2	2	2	3	2	1	2	N
4e2s_A	UGY	1	2	1	1	2	4	1	1	7
4erc_A	VN4	1	1	1	1	N	6	9	1	N
4f0d_A	3AB	4	4	N	N	10	N	1	4	3
4fbl_A	SPD	1	1	1	1	2	1	2	1	5
4fq5_A	MAE	1	1	1	2	3	N	10	1	N
4gjz_A	AKG	14	18	N	N	4	17	1	18	1
4hdr_A	DMD	1	1	1	1	1	43	6	1	2
4hkg_A	MRD	1	1	1	1	1	2	1	1	1
4hwh_A	MLA	3	1	1	1	N	1	1	1	2
4j25_A	OGA	1	1	1	1	1	1	1	1	3
4jdf_A	SPD	1	1	1	1	1	1	1	1	1
4k91_A	SIN	3	3	2	2	11	2	1	3	3
4lit_A	AKG	1	2	1	1	3	1	1	1	1
4lwl_A	ACT	2	1	2	2	1	1	1	1	3
4m25_A	AKG	N	N	5	1	N	N	6	N	1
4m4g_A	PEG	1	1	2	2	1	4	1	1	8
4mx6_A	SIN	1	2	5	N	3	22	1	2	N
4npl_A	AKG	1	1	1	1	2	N	1	1	7
4nrp_A	OGA	1	1	1	1	1	49	1	1	1
4ov4_A	KIV	1	2	2	2	3	N	1	2	3
4ptn_A	GXV	1	1	1	1	1	1	1	1	2
4qhq_A	PEG	1	2	2	1	1	3	1	1	2
4qu2_A	AKG	1	2	N	N	N	N	1	1	3
4r17_K	3K4	1	1	1	1	5	N	3	1	1
4tgl_A	DEP	1	1	1	1	N	1	1	1	1
4uqw_A	BEN	21	18	5	1	N	9	4	15	N
4wwq_A	MLA	3	4	1	1	N	2	2	4	10
4xdq_A	BEZ	1	1	1	1	1	12	1	1	1
4xez_A	THJ	1	3	1	N	N	1	1	1	N

4yi7_A	BE2	1	1	1	1	3	1	1	1	2
4yn5_A	CAC	1	1	1	1	4	N	1	1	2
4z67_A	MLT	1	1	1	1	1	1	1	1	1
4zch_A	144	3	4	N	N	6	7	1	5	3
4zxa_W	H8N	2	3	2	2	2	N	1	2	N
5a0u_A	CHT	8	9	N	11	18	26	1	5	N
5c5t_A	AKG	1	1	1	1	3	2	1	1	3
5cge_A	51F	16	26	5	N	N	31	N	17	4
5cta_A	BM0	11	2	3	3	2	3	2	11	4
5dmp_A	VN4	1	1	1	1	1	4	1	1	1
5i8t_A	LAC	1	1	1	1	1	2	1	1	1
5im2_A	BEZ	1	1	2	3	6	1	2	1	9
5j39_A	CAC	1	1	2	1	3	3	1	2	5

Table S6. Detailed information of small-volume ligand-binding site prediction using the MF-PLB, PLB, Ligsite-csc, MPK2, ConCavity, Surfnet, Q-SiteFinder, LISE and SiteHound. The numbers in the table reflect the ranking of the actual ligand-binding site, and "N" indicates that the ligand-binding site was not found using the method.

PDB_Chain	Ligand	MF-PLB	Ligsite-csc	MPK2	ConCavity	Q-SiteFinder	Surfnet	LISE	PLB	SiteHound
1ab8AB	FOK	1	1	1	1	1	1	1	1	1
1aj8AB	COA, CIT	1	1	1	1	1	2	1	1	1
1b57AB	PGH	2	3	3	3	2	N	1	2	10
1b8gAB	PLP	N	N	N	N	9	N	1	N	N
1bcpKL	ATP	1	1	1	1	1	39	1	1	1
1bh5AB	GTX	1	1	1	1	3	N	1	1	1
1buiAC	0GJ	2	2	5	2	7	N	2	2	5
1bw0AB	LLP	1	1	1	2	1	1	1	1	1
1byeCD	ATA	N	N	5	N	4	N	1	N	6
1c7gAC	PLP	N	N	N	N	4	N	3	N	N
1c7oCD	PPG	22	52	3	N	2	N	1	20	5
1chmAB	CMS	3	9	N	N	8	15	1	3	N
1csmAB	TRP	1	1	2	N	1	N	3	1	5
1d2fAB	PLP	N	N	2	N	2	N	1	N	5
1dcpEF	HBI	1	1	1	1	1	2	1	2	1
1dlmAB	LIO	1	1	1	1	1	1	N	1	4
1dqaCD	COA, NAP, MAH	1	1	1	1	1	N	1	1	1
1dqxCD	BMP	1	1	1	1	1	N	1	1	1
1dtyAB	PLP	20	48	N	N	N	N	4	37	N
1dugAB	GSH	N	N	N	N	1	N	3	N	1
1e0jBC	ANP	1	1	1	1	2	N	1	1	1
1e5fAB	PLP	3	7	5	N	1	N	1	3	N
1ecmAB	TSA	1	1	1	1	3	1	1	1	1
1eixAB	BMQ	1	1	1	1	1	2	1	2	4
1ewjEF	BLM	1	1	2	1	1	2	1	2	1
1f34AB	SEP,M	40	44	N	N	1	N	1	45	3

	PD									
1f5vAB	FMN	1	1	1	1	1	1	1	1	1
1fc4AB	AKB, PLP	1	1	2	N	3	N	1	1	4
1ft9AB	HEM	1	3	1	1	1	N	4	1	2
1g5cAB	EPE	3	4	7	2	5	5	5	2	5
1g64AB	B12,ATP	1	1	1	1	1	1	1	1	N
1grnAB	GDP,AF3	N	N	2	N	1	1	2	N	3
1gufAB	NDP	1	1	1	1	1	N	1	1	1
1guqCD	UPG	1	2	3	2	3	N	1	2	1
1gyxAB	BEZ,EPE	2	1	1	2	1	2	1	1	1
1gztAB	FUC	1	3	5	1	N	1	1	2	1
1he1AC	GDP,AF3	1	1	4	1	1	1	1	1	1
1hkvAB	LYS,PLP	1	1	1	1	1	N	1	1	3
1hw5AB	CMP	5	7	2	2	5	1	6	5	3
1i69AB	BEZ	1	1	1	1	N	1	1	1	1
1idtAB	FMN, CB1	1	1	1	1	1	N	1	1	2
1ig3AB	VIB	1	1	1	1	1	N	1	1	1
1iugAB	LLP	45	48	N	N	2	N	2	41	1
1iyiAD	GSH	N	N	6	N	1	N	1	N	4
1j2gAB	AZA	N	N	N	2	9	N	1	N	1
1j6wAB	MET	1	1	1	1	1	1	1	1	1
1jlrBD	GTP	3	5	6	N	5	4	5	4	2
1js6AB	PLP	N	N	1	1	1	N	1	N	2
1jwbBD	AMP	N	N	N	N	4	N	1	N	1
1k0cAB	GTB	1	1	1	1	1	2	2	1	1

1knyAB	APC, KAN	1	1	1	1	1	1	1	1	1
1kpeAB	ADW	1	2	1	1	1	1	1	1	1
1lluAB	NAD	1	1	1	1	2	N	2	1	3
1m32AB	PLP,POA	9	29	5	2	2	N	1	20	4
1mkaAB	DAC	1	1	1	1	1	2	1	1	3
1n2fAB	DTT	1	1	1	1	1	3	1	1	1
1nbfBC	GLZ	32	54	N	N	N	23	N	39	N
1ni4AD	TPP	1	1	1	1	1	1	1	1	8
1nkiAB	PPF	1	1	1	1	1	2	1	1	2
1nw4CD	IMH,IPA	1	1	1	1	1	N	1	1	1
1nzcAB	TDX	1	1	1	1	1	3	1	1	1
1oe8AB	GSH	N	N	N	N	15	N	2	N	2
1ohvAB	PLP,FES	38	51	2	1	1	1	3	36	8
1osyAB	ACE	24	24	N	N	N	N	N	23	N
1p4aCD	PCP	10	19	3	2	2	N	4	21	2
1q0kBE	THJ	1	1	1	1	N	N	1	1	2
1q4tAB	4CO	1	1	1	1	2	N	2	1	1
1q5hBC	DUD	2	3	5	4	5	4	4	5	2
1qapAB	NTM	2	2	1	1	3	N	1	2	1
1qk3AB	5GP	1	3	7	3	4	N	10	2	7
1qqjAB	CAC	N	N	3	3	1	N	N	N	1
1qu4AB	PLP	2	3	1	1	5	N	1	2	3
1r8sAE	GDP,SO3	1	2	3	1	1	N	2	1	N
1re5CD	CIT	55	56	N	N	42	N	N	95	N
1rl8AB	RIT	1	1	1	1	1	1	3	1	1
1rozAB	NAD	12	8	N	N	13	4	N	15	N
1s0yAD	MLA	1	3	2	N	N	1	2	2	N

1s63AB	SUC,FPP,778	1	1	1	1	1	N	1	1	8
1sc3AB	MLI	1	6	1	1	13	N	1	3	3
1so3AB	TX4	1	1	1	1	3	N	1	1	1
1sqsAB	TLA	N	N	N	N	12	N	3	N	3
1ss4AB	CIT,GSH	14	1	1	1	N	27	1	14	8
1stfEI	CCS	4	3	7	2	14	N	7	2	N
1sz2AB	BGC	1	1	1	1	21	N	1	1	2
1sz3AB	GNP	2	3	4	3	2	N	1	3	3
1t0iAB	FMN	1	1	1	1	1	1	1	1	1
1t0lCD	NAP,ICT	1	1	1	1	1	N	1	1	2
1t5bAB	FMN	1	1	1	1	1	1	1	1	1
1tu6AB	FSP	1	1	1	1	1	N	1	1	1
1ty9AB	FMN	1	1	1	1	1	N	1	1	1
1u08AB	PLP	N	N	6	N	1	N	2	N	N
1v3vAB	NAP,5OP	1	1	1	1	1	1	1	1	1
1vfsAB	KCX,DCS	2	2	2	2	1	1	1	2	3
1vgqAB	CAO	1	1	1	1	1	2	1	1	2
1vhzAB	APR	N	N	N	N	4	N	2	N	4
1vi0AB	DCC	1	1	1	1	1	1	1	1	1
1vp4AB	PLP,UNL	1	1	1	1	1	N	1	1	1
1w2yAB	DUN	N	N	3	N	3	N	1	N	1
1w98AB	TPO	N	N	10	3	21	N	5	N	10
1wgtAB	PCA	28	23	N	N	N	N	N	30	N
1wkqAB	IMD	2	6	8	2	5	10	1	1	N
1wliAB	FMN	1	1	1	1	1	1	2	1	1
1wofAB	I12	3	3	5	2	3	N	6	3	1

1wyuGH	PLP	N	N	N	N	5	N	1	N	N
1x9hAB	F6R	3	3	7	2	6	N	7	3	3
1xa4AB	BTB,COA	1	1	1	1	4	N	1	1	3
1xd3AB	GVE	1	1	N	N	N	N	2	1	4
1xetAB	3IO	1	1	1	1	N	3	4	2	3
1xrkAB	BLM	1	1	2	1	1	N	1	1	1
1y0gCD	8PP	1	1	2	1	2	2	1	1	2
1y7pAC	RIP	3	3	3	3	17	1	1	3	10
1yaaCD	MAE, PLP	1	3	8	3	2	N	1	1	7
1yb5AB	NAP	1	1	1	1	1	N	5	1	1
1yiyAB	PMP	N	N	N	N	10	N	1	N	1
1yliAB	COA	1	1	1	1	1	1	6	1	1
1ypqAB	DIO	1	1	1	1	1	2	1	1	1
1yqdAB	NAP,DTT	1	1	2	1	4	N	1	1	1
1ytaAB	FLC	1	1	3	1	5	4	6	1	4
1yukAB	NDG	N	N	N	N	N	43	N	N	N
1yzxAB	GSF	1	1	1	1	1	1	1	1	2
1z2iAB	NAD	1	1	1	1	1	1	1	1	1
1zhhAB	NHE	1	40	3	2	9	N	3	1	8
1zosCD	MTM	1	1	1	2	2	4	1	1	1
2a87AB	FAD, NAP	32	41	4	1	1	N	2	23	2
2af6BC	FAD,BRU	4	5	4	1	3	1	1	8	1
2ag1CD	TPP	1	1	1	1	1	N	N	1	1
2arvAB	1PG	5	5	7	N	4	11	1	4	7
2b5aAB	ACY	N	N	N	N	N	11	4	N	N
2b67CD	FMN	1	1	1	1	1	N	2	1	1
2betCD	DEZ	1	1	1	1	1	N	1	1	2

2bnnAB	FCN	1	1	2	1	11	N	1	1	8
2c0rAB	PLP	17	37	N	N	2	N	2	37	9
2cg5AB	COA	1	1	1	1	1	1	1	1	2
2ch1AD	PLP	3	5	N	N	4	N	1	5	N
2czeAB	U5P,CIT	1	1	1	1	1	2	1	1	1
2f62AB	12M	1	1	1	1	1	N	3	1	1
2f7sAB	GDP	2	4	5	N	19	3	3	8	N
2f98CD	NGV	N	N	2	N	1	N	1	N	2
2fm1AD	LLP	1	1	1	1	1	1	1	1	1
2fnuAB	PMP,UD1	1	1	1	1	1	2	1	1	1
2freAB	FMN	1	1	1	1	1	N	1	1	1
2fxaAB	1PE,P6G	2	2	2	2	2	N	1	2	2
2g9zAB	VNP	1	1	1	1	4	N	1	1	1
2gb3EF	LLP	29	59	N	N	3	N	1	25	7
2gf6AB	COA	1	1	1	1	2	N	1	1	1
3proAC	AES	3	2	2	2	1	N	2	3	4
4ecaAC	AEI	2	2	3	2	11	41	5	1	N
6cscAB	COF,CIT	1	1	1	1	1	1	1	1	1
6pfkAB	PGA	1	6	8	3	5	6	2	5	7

Table S7. Detailed prediction results of ligand-binding sites on protein-protein interfaces using the MF-PLB, PLB, Ligsite-csc, MPK2, ConCavity, Surfnet, Q-SiteFinder, LISE and SiteHound. The numbers in the table reflect the ranking of the actual ligand-binding site, and “N” indicates that the ligand-binding site was not found using the method.
